# The social validity of a national assessment centre for selection into general practice training

**DOI:** 10.1186/s12909-014-0261-6

**Published:** 2014-12-21

**Authors:** Annette Burgess, Chris Roberts, Tyler Clark, Karyn Mossman

**Affiliations:** Sydney Medical School-Central, The University of Sydney, Building 63, level 4, Royal Prince Alfred Hospital, Missenden Road, Camperdown, NSW 2050 New South Wales Australia; Sydney Medical School-Northern, The University of Sydney, New South Wales, Australia; Sydney Medical School, Office of Medical Education, The University of Sydney, New South Wales, Australia

**Keywords:** Social validity, Postgraduate medical training, Assessment centre, Multiple-mini-interview, Situational judgement test, General practice, Selection, Admissions

## Abstract

**Background:**

Internationally, recruiting the best candidates is central to the success of postgraduate training programs and the quality of the medical workforce. So far there has been little theoretically informed research considering selection systems from the perspective of the candidates. We explored candidates’ perception of the fairness of a National Assessment Centre (NAC) approach for selection into Australian general practice training, where candidates were assessed by a Multiple Mini Interview (MMI) and a written Situational Judgment Test (SJT), for suitability to undertake general practice (GP) training.

**Methods:**

In 2013, 1,930 medical practitioners, who were eligible to work in Australia attended one of 14 NACs in each of 5 states and 2 territories. A survey was distributed to each candidate at the conclusion of their assessment, which included open-ended questions aimed at eliciting candidates’ perceived benefits and challenges of the selection process. A framework analysis was informed by the theoretical lens of Social Validity Theory.

**Results:**

Qualitative data was available from 46% (n = 886/1,930) of candidates, who found the NAC experience fair and informative for their training and career goals, but wanted to be provided with more information in preparation. Candidates valued being able to communicate their skills during the MMI, but found some difficulty in interpreting the questions. A significant minority had concerns that a lack of relevant GP experience may inhibit their performance. Candidates also expressed concerns about the time limits within the written paper, particularly if English was not their first language. They also expressed a desire for formative feedback during the interview process.

**Conclusion:**

During any job selection process, not only is the organisation assessing the candidates, but the candidates are also assessing the organisation. However, a focus on the candidate experience throughout an organisation’s selection process may provide benefits to both candidates and the organisation, regardless of whether or not candidates secured the job. Social Validity Theory is a useful addition to the methods for demonstrating the reasonableness of any selection system.

## Background

Internationally, the process of recruiting and selecting the best candidates is central to the success of postgraduate training programs and the quality of the medical workforce. A key goal is to predict which candidates will go on and become able doctors, and reject those who are likely to perform poorly in future practice due to issues of professional behaviour as well as lack of clinical knowledge and skills [1]. However, from a candidate’s perspective selection systems are high stakes and have a major impact on their attitude to both the employer and the proposed place of work [2]. Research has tended to privilege the perspective of the employers. However, it is critical for healthcare education and training organisations to consider selection systems from the perspective of both the employer and the job candidate.

There are a number of factors in the organisational literature that evidence how the selection experience can influence a candidate’s perception of the organisation and the position they are applying for [2]. Changing and starting new jobs is ranked in the top 40 of life’s most stressful events [3]. It has been predicted that a candidate’s perception of the fairness of a selection system can influence their future attitudes, intentions, and behaviours in the workplace. Candidates who become disgruntled by the process may develop a negative opinion of the organisation, communicating this to other professionals, having direct implications on organisational reputation [4,5]. Negative reactions to the application process may also influence the attitudes and performance of candidates within the selection process [6], with those who perceive it as problematic being reluctant to fully participate and engage [7]. When candidates are highly qualified, with various job choices, they are less likely to proceed with a poorly conceived selection process, and consequently, organisations may lose the most outstanding candidates. Inappropriate selection procedures may cause candidates to dispute selection outcomes [8], to the point of legal redress. It has also been suggested that negative experiences for candidates during the selection process can have a detrimental effect on the candidates’ wellbeing [9,10]. It is therefore imperative to understand the mechanisms by which candidates cognitively assess selection measures so that institutions are able to refine their processes to attract and retain the most qualified candidates.

Postgraduate healthcare selection procedures generally set a minimum standard of clinical competence and personal and professional values, that are expected of entry-level professionals. These are required to be acceptable to a range of interested stakeholders, including universities, government, health service employers, the professional colleges, and the wider community. Typically, selection admission committees develop a ranking list of candidates, which descends in merit order until allocable places are exhausted. The traditional system of ranking candidates consisted of a mixture of application forms, traditional panel interviews, personal statements, and references [11]. Internationally, there have been moves to develop selection procedures which are much more robust in terms of their underlying assessable constructs, psychometrics, fairness and defensibility. The term assessment centre [12] has been used to refer to a model where candidates are required to attend a venue to undertake more than one assessment for the purpose of selection into postgraduate training. Internationally, such assessment centres have used a range of formats, both written and observed, including situational judgement tests (SJT) [13], clinical problem solving tests (CPST) [12], low and high fidelity simulations [14], and the multiple-mini-interview (MMI) [15].

Previous research has demonstrated that candidates are not passive within the selection process, but actively seek opportunities to enhance their chances of presenting a favourable impression, even within short multiple interviews. This is consistent with the literature on the use of impression management. For example, in the high-stakes setting of selection into medical school, students actively tried to shape the impressions that their interviewers might make of them unrelated to the particular set of questions being asked at that interview station [16].

Inevitably, the process of candidate selection involves a measure of differentiation between candidates in order to choose the best candidate for the job, leaving a number of candidates disappointed at the outcome. Any assessment procedure should be free of bias, so that candidates of equal ability are not discriminated against. However, there are widely acknowledged issues of potential biases in selection on the grounds of gender, age, culture and ethnicity. For example, in a study exploring the relative importance attached to various perceived aspects of fairness in personnel selection in a North American setting, ethnicity analyses indicated different ethnic groups emphasised different characteristics in inferring fairness [17]. Potential biases in selection might also impact on the cultural diversity of the workforce. Concerns have been raised regarding entry into general practice in the UK, for black and ethnic minorities were more likely to fail the barrier assessment of GP training than white doctors [18]. Organisations are also encouraged to plan around cultural appropriateness of selection procedures to reduce potential litigation as perceived unfairness is a major cause of costly litigation within selection [17].

A number of frameworks have been proposed to predict, understand and influence job candidates’ reactions to the selection system, and the extent to which that differentiation is based on capability and not on extraneous factors such as, age, gender, and culture. In the field of organisational psychology, frameworks for considering candidate perception of selection processes have primarily revolved around organisational justice theory. There are broadly two differing sets of justice rules that apply in the context of selection. Procedural justice rules are based on the fairness of how decisions are made, while distributive justice rules centre around candidates’ perception of fairness of outcomes [2]. It is common to both rule sets of organisational justice to ensure that a candidate should feel that they have gone through a fair recruitment and selection process.

Within postgraduate specialty training in the UK, Patterson et al. have used a model of organisational justice theory to evaluate candidate reactions about the selection processes used in that setting [19]. Candidates consistently viewed the high-fidelity selection methods, as more job-related and fairer. The authors advised recruiters to systematically compare perceptions of the fairness and job relevance of the various selection methods they were using in their own setting. Within the broader selection literature, candidates’ preferences for selection techniques have been shown to be interviews, followed by work samples, resumes and tests [20,21].

We have argued that properly conducted selection systems are in the best interest of both the candidate and the organisation [22]. However, so far there has been little theoretically informed research in postgraduate specialty training to determine to what extent candidate reactions are well aligned with a selection process whose purpose is to identify the best candidates for the positions available.

### Research context

The details of our research context are published elsewhere [23,24], and we summarise here. The Australian General Practice and Training (AGPT) introduced a National Assessment Centre (NAC) approach to General Practice (GP) training in 2011. In 2013, 17 regional training providers (RTPs) alongside GPET, ran 14 NACs across Australia. Two assessments were used in the NAC for candidates who were eligible to work in Australia; the observed multiple mini-interviews (MMI) and the written situational judgement test (SJT).

The MMI is an interview format that uses many short independent assessments, each assessed by a single previously trained interviewer. They have been used to assess non-cognitive characteristics of postgraduate medical trainees in the United Kingdom (UK) [25], Canada [15] and Australia [23]. Early findings suggest that the MMI is a useful format for the selection of junior doctors into speciality training.

SJTs are a written assessment format also used to test non-cognitive characteristics. They involve authentic, hypothetical scenarios requiring the individual to identify the most appropriate response or to rank the responses in the order they feel is most effective. Evidence supporting the validity and reliability of the SJT as a shortlisting tool in postgraduate selection has prompted their introduction into the selection process of several medical specialities within the UK. As a relatively low-resource assessment, SJTs are claimed to be a cost-efficient methodology compared with resource intensive assessments of non-cognitive attributes [13] like the MMI.

The NAC assessments had been blueprinted against the expected competencies of entry-level registrars in six domains of practice set out by the two professional colleges in Australia (the Royal Australian College of General Practice and the Australian College of Rural and Remote Medicine). These domains included communication and interpersonal skills, clinical reasoning, analytical/problem solving skills; organisational/management skills; sense of vocation/motivation; personal attributes (including the capacity for self-reflection and awareness of the impact of cultural issues on delivery of primary health care) and professional/ethical attributes.

In 2013, candidates attended one of 14 NACs where they sat a 100 minute 50-question Situational Judgement Test (SJT), and a six station Multiple Mini Interview (MMI). Candidates were assigned an AGPT ranking band score based on MMI and SJT score on a 50–50 split. Those candidates with sufficient NAC total score (combined SJT and MMI scores) were passed to their preferred Regional Training Provider (RTP). Upon review of candidate scores, RTPs had the option of accepting candidates based on their scores alone or conducting an additional round of interviews, and reviewing referee reports if desired. Data for the RTP led process was not part of the NAC process nor this particular research.

MMI marking criteria for each non-cognitive domain (for example, vocation/motivation), included the scope of the desired behaviours (for example, enthusiasm for a career in general practice or dealing with an angry patient), which was to be marked using a seven point rating scale. This ranged from 1 (unsuitable/does not meet criterion) to 4 (meets criterion) through to 7 (meets criterion to superior degree). For each anchor, descriptors were provided to indicate examples of the ways in which candidates might meet the criteria in the interview. Interviewers were encouraged to write sufficient notes on the marking sheet to justify their decision particularly if the candidates did not meet the criteria.

### Theoretical research framework

Typically a major concern of any assessment system, is its validity, and there are many models that are widely used in medical education, for example, Van Der Vleuten’s utility index which explores reliability, validity, educational impact, acceptability, feasibility and cost [26]. A recognised limitation of this model is that test takers are merely asked to what extent they find a given assessment acceptable. Accordingly there has been interest in evidencing additional forms of validity to demonstrate the utility of a selection system. Social Validity was originally described by Wolf (1978) to inform the development of better systems and measures to determine whether society was accomplishing the objectives of any particular social intervention [27]. In particular, the social significance of the goals, the social appropriateness of the procedures, and the social importance of the effects of the intervention. Social Validity in the context of selection was further developed by Schuler (1993) to consider the social impact of selection technology on participants in the selection process [28]. Schuler’s theory focused on the extent to which candidates developed both positive and negative perceptions of fairness in the way they experienced the selection system. A Social validity framework, according to Schuler (1993), has four key features that represent a fair and acceptable process to candidates [28] and invites the individual candidate to reflect on how they are personally impacted during the selection process.

1. Provision of relevant information regarding the job and the organisation.
2. Opportunity to practice and display relevant knowledge and skills.
3. Transparency of the selection process and selection tools.
4. Provision of feedback regarding results.

For our study we posed the research question: What are the underlying factors which influence candidates’ perceptions of the fairness of a national assessment centre approach for selection into general practice training using the theoretical perspective of Schuler’s (1993) Social Validity Theory [28].

## Methods

In 2013, 1,930 medical practitioners who were eligible to work in Australia applied to AGPT. Of these candidates, 1093 (56.6%) were born outside of Australia, and 606 (31.4%) obtained their primary medical qualification outside of Australia. Candidates attended one of 14 NACs, in each of 5 states and 2 territories nationwide. As part of a systematic evaluation of the process, an anonymous candidate questionnaire was distributed to each candidate immediately following completion of the SJT and MMI at the NAC. The questionnaire included open-ended questions aimed at eliciting candidates’ perceived benefits and the most challenging aspects of being assessed by way of an NAC.

A thematic analysis of the qualitative data was done using Framework Analysis [29]. Coding focused on the socio-cultural influences of the experiences, interactions, and beliefs that influenced the candidates. Whilst this was initially done inductively by all four authors, in subsequent analysis of data from the perspective of candidate fairness, we noted that the emergent themes from the inductive analysis resonated with key constructs within Social Validity Theory [28]. At this point the authors discussed the value of using the theory as the conceptual framework for this paper and subsequently developed a thematic framework, which was applied to a portion of the dataset by three authors to establish its trustworthiness, and to check for new and emerging issues of importance that would extend the analysis. Subsequently the first author coded all of the data, in order to identify recurrent themes and subthemes in the data [30]. Once data had been coded and categorized deductively into themes, the data within each theme were quantified in order to measure thematic prevalence [29].

### Ethical considerations

The University of Sydney Human Research Ethics Committee approved the research. All candidates were reassured that data was strictly de-identified to protect participant privacy.

## Results

Data was available from 886/1,930 meaning that nearly half (46%) of all candidates provided constructive comments about the NAC. Candidates’ responses to open ended questions regarding the most beneficial and most challenging aspects of being assessed via the NAC selection process are summarized in Table 1, and were mapped to the conceptual framework of Social Validity Theory [28].

**Table 1:** **Candidate responses to open ended questions regarding their perceptions of the selection process (n = 886)**

**1. Provision of relevant information regarding the job and the organisation.**					
**Positive Features**			**Negative Features**		
71 (8%)	Candidates’ experience of the NAC provided insight and understanding into the requirements of general practice training.	*“I received a wider knowledge of common issues in GP practice in Australia”.*	58 (7%)	Candidates perceived there to be inadequate information provided prior to attendance at the NAC.	*“There was a lack of references available or a guide to the type of preparation required”.*
					*“Little information beforehand re. format of the day”.*
		*“Very real scenarios allowing me to know what to expect from GP. I now have an understanding that some scenarios require quick decision making skills”.*			*“Little more material for preparation especially SJT as quite a specific MCQ style not everyone would be familiar with”.*
**2. Opportunity to participate and display relevant knowledge and skills.**					
**Positive Features**			**Negative Features**		
384 (43%)	Candidates valued the face to face interaction with interviewers during the MMI and the opportunity to demonstrate their experience and skills.	*“Face to face MMI was good as it provides an opportunity to explain answers and discuss”.*	380 (43%)	Candidates expressed concern about inadequate time to complete the SJT and difficulty in interpreting questions.	*“English is not my first language so there was not enough time in the SJT to read lengthy paragraphs”.*
		*“Able to highlight past skills and experience”.*			SJT questions were *“incredibly vague”, “ambiguous”, and “confusing”.*
		*“….allows me to show clear clinical judgement and reasoning”.*			*“SJT – difficult because no opportunity to justify answers”.*
62 (7%)	Candidates valued the opportunity for assessment of areas other than knowledge and clinical skills.	*“It assessed me on different skills both personal and professional”.*	83 (9%)	Candidates found it difficult to respond to the MMI questions due to lack of relevant experience.	*“Very GP focused in some ways. Not all of us have had GP rotations yet!”*
		*“Allows me to speak for myself and show my personality traits, not just on paper”.*			*“Not yet having experience in GP and many of the MMI questions referred to a GP scenario”.*
**3. Transparency of the selection process and the selection tools.**					
**Positive Features**			**Negative Features**		
186 (21%)	Generally, the candidates found the NAC selection process to be fair and equitable.	*“Fair across all criteria and all states”.*	189 (21%)	Candidates had difficulty in interpreting the MMI questions – or the style of the interviewer. They were unsure what some questions were asking, and needed more guidance.	“I *had difficulty with clarification of questions during the MMI”.*
		*“It did not appear to matter the level at which one is working ie. Intern vs more senior”.*			*“For one of the MMI stations the examiner started to use closed questions almost immediately without letting me explore the situation. Given that, I felt thrown off guard”.*
		*“SJT is anonymous - takes out role of interviewer bias when it comes to candidates”.*			*“Some questions were not very clear, so it’s hard to address solutions”.*
**4. Provision of feedback regarding results**					
**Positive Features**			**Negative Features**		
32 (4%)	Although applicants felt that the process provided a suitable ranking method to inform RTPs, they reflected on expectations about feedback on their performance.	*“Allows ranking for RTP's and finds suitability”.*	52 (6%)	Candidates found it difficult to gauge their own performance. They expressed a desire for some kind of immediate, formative individual feedback.	*“The MMI stations were vague with no immediate feedback.”*
		*“Allows differentiation between candidates”.*			*“Interviews are always stressful - hard when you don't get any feedback.”*
		*“Get an idea about the issues faced at the GP practices. Also get feedback for further improvements in areas which you are not familiar”.*			*“No feedback regarding our performance (often subtle feedback is given even at the most formal college interview processes)”.*
		*“Face to face examination allows assessment of communication skills and immediate feedback from examiner, to ensure complete answering of questions”*			

Inside Table 1, percentage scores do not necessarily total 100% as some respondent expressed more than one theme in a given response.

### Provision of relevant information regarding the job and the organisation

A detailed handbook explaining the selection and training was provided by the AGPT and was downloadable from their website. However, our data suggest that candidates wanted more specific types of information in preparation for attendance at the NAC. For example, although candidates thought the SJT and MMI sample questions provided by GPET prior to these assessments were relevant, they would have liked access to a preparation guide and additional example questions, with 58/886 (7%) of survey respondents echoing the sentiment, “*You didn’t have an idea of what to expect until you got here”.*

Interestingly, 71/886 (8%) of respondents found their experience at the NAC provided them with insight and understanding of what is required to work in general practice, and that the experience of the selection process itself would be helpful for them to apply again in the future if they were unsuccessful. This supports the idea that more detailed information regarding the requirements of a general practice registrar position should be provided to the candidates that gives a realistic preview of the job they are going into, and is thought to be related to higher performance and lower attrition rates [31]. However it also suggests that simply providing passive information is insufficient. Rather candidates are learning about general practice through the selection process as a form of situated learning [32]. Thought needs to be given to the educational design of candidate orientation to the selection process, information regarding a career in general practice, the materials which support understanding of the candidate pathway through the selection process, and supporting materials and guidance on the types of feedback available, during the process and on notification of the outcome of the application.

### Opportunity to participate and display relevant knowledge and skills

Candidate perceptions of biases within interview questions were apparent depending upon candidates’ experience, culture, or whether English was a first language. Some candidates found it difficult to respond to the MMI questions due to their lack of relevant experience in an Australian general practice setting, with 83/886 (9%) noting difficulty in answering some questions due to their lack of relative experience. It is possible that less experienced, and also international medical graduates, some of who were trained as specialists in their country of origin, may perceive that there are biases within the questions. Local candidates may be better equipped to answer the MMI questions because of their relevant cultural experience. For example, an international medical graduate may lack the experience of local candidates that would provide insight and understanding into the practices of local GPs. Some found it difficult to respond to the MMI when they had not had experience practicing as a GP, *“Very GP focused in some ways. Not all of us have had GP rotations yet*”. For example, interns are only hospital focussed, not hospital and community focussed. It follows that candidates may have underlying concerns that they are being scored by interviewers for their readiness to practice, that is their ability to commence work, rather than as was intended by the selection process, for their trainability.

Although most candidates did not comment on the SJT, a significant minority of candidates mentioned that the SJT questions were more difficult to respond to than the MMI questions, with 156/886 (18%) commenting that they found the SJT questions to be *“vague”* and *“confusing”,* suggesting that SJT questions were not representative of the candidates’ broader experiences or their level of experience.

Candidates [380/886 (43%) of survey respondents] also commented on their difficulty in responding to the SJT questions because of their ambiguous nature, and in completing the SJT in the given timeframe, particularly if English was not their first language. English difficulties have previously been reported in regards to entrance exam outcomes for IMGs [33].

It appeared that candidates placed greater value on the MMI questions and the face to face opportunity for interactions with interviewers, with 384/886 (43%) of survey respondents commenting that the interviews allowed them to *“explain and discuss answers”, and “highlight past skills and experience”.* Candidates felt that they were able to display relevant attributes other than knowledge and clinical skills, with 62/886 (7%) of survey respondents commenting that they were assessed *“on different skills both personal and professional”.*

### Transparency of the selection process and the selection tools

Candidates generally found the national assessment process to be fair, with 186/886 (21%) of survey respondents conveying that they considered it to be *“fair across all criteria and all states” and “standardised”.* International Medical Graduates mostly perceived the process fair. Their concerns were largely about their own experience, and if relevant, their command of English language.

Candidates [189/886 (21%) of survey respondents] found the MMI questions quite broad, and commented on their difficulty in interpretation, and development of appropriate responses. Candidates would have liked more guidance and prompting from the interviewers in order to help them to stay on track. This resonates with findings elsewhere advocating standardised, structured prompting within interviews, and the need of adequate interviewer training [34].

### Provision of feedback regarding results

At the time of survey distribution and completion, candidates appeared more concerned with immediate formative feedback rather than summative feedback. Many candidates expressed a desire for formative feedback, with 52/886 (6%) commenting, *“The MMI stations were vague with no immediate feedback”.* Considering that performance feedback provided to RPTs was necessary to inform ranking, they did not appear to have considered whether the summative feedback would be used to inform their future training and practice if successful, or to improve future NAC performance if unsuccessful. Provision of feedback can offer a valuable method to enrich the candidates’ performance and learning experience. By providing candidates with feedback, it is possible that the gap between actual and desired performance in the future can be narrowed [35]. However, the perception of the quality of feedback is important in eliciting a positive attitude towards change [36]. It is therefore important that if feedback is to be given, consideration is given to the quality and value of both formative and summative feedback that could potentially be provided to the candidates.

## Discussion

Our data provides evidence, that a national selection centre for selecting doctors to enter general practice has a modest degree of social validity [28]. In general, the NAC process was perceived by candidates to be fair and transparent. Our data show that candidates found the NAC experience itself informative about a job as a GP registrar and about a career in general practice, but wanted to be provided with more information in preparation for their attendance at the NAC. Candidates valued being able to convey a range of skills and experience during the MMIs, but found some difficulty in interpreting the questions, and were concerned that a lack of relevant experience in general practice might have inhibited their performance. However, candidates would particularly like to be provided with more information about the MMI prior to the assessment. They would like more guidance and prompts to answer the questions, and more opportunity to demonstrate their experiences. Candidates also expressed concerns about the time limits within the SJT, particularly if English was not their first language. Candidates appeared to have little expectation regarding feedback about the decision to progress or not to the next round at the RTPs. However, candidates did express concerns about their ability to gauge their own performance during the MMI interview process, and would appreciate more immediate feedback.

Social Validity Theory [28] offers a useful framework to assess candidates’ perceptions of their experience during the selection process conducted by AGPT and the RTPs. Our data correlates with the Schuler’s (1993) contention that the first three elements are most highly regarded by candidates; that is information, participation and transparency [28]. That candidates appeared to be less concerned with the element of feedback resonated with the finding of others [28]. We now consider each element in more detail.

### Provision of relevant job related information

Provision of information refers to the degree to which the candidate perceives the information provided by the organisation as useful [28]. Maintaining clear and open communication is important in ensuring that candidates feel they have been treated fairly and humanly [6,2,37]. Support to candidates can be provided throughout the recruitment process by ensuring candidates are able to ask questions and obtain information as required [6,37]. Results from our study suggest that the need for provision of information to candidates prior to the assessment activities should not be underestimated. Furthermore, our results validate the need for a selection method that is closely aligned with the expected competencies of the job for which it is selecting.

### Opportunity to participate

Opportunity to participate refers to the extent to which the candidates feel they have the opportunity to display their knowledge and skills to their potential employer [6,37]. If candidates feel that they have been assessed on criteria that are irrelevant to the job, they feel they have forgone an opportunity to do this [6]. Our results suggest that generally, candidates appreciated the inclusion of MMIs in the assessment process, as it allowed them to portray their personal and professional attributes. However, it was generally felt that candidates with fewer opportunities to gain Australian-specific general practice experience, such as interns; medical practitioners without general practice training; and International Medical Graduates, may perceive some of the experience based questions posed within both the SJT and MMI questions as unfair. This is important given international concerns that international medical graduates and locally based ethnic minorities may be discriminated against in assessments for the right to practice [18]. It is likely that selection organisations will need to scrutinise their data using a range of methods to assure that processes are culturally appropriate and focus on candidate ability to do a complete job [17].

### Transparency

Transparency refers to the extent to which the candidates feel the selection method is clear and standardized for all participants. Openness in terms of selection process and procedures can help to increase the candidates’ perception of fairness as well as organisational attractiveness [37]. Candidates want a level playing field to ensure an opportunity to be assessed fairly [10,38]. The organisation and implementation of MMIs and SJTs at the NACs was standardized across all sites, and candidates generally found the application process to be fair and equitable. AGPT makes the point that the purpose of the NAC is to provide a ranked list of competent candidates for local RTPs, who then make the placement with the local GP supervisors in a locally determined way. However, it is noteworthy that beyond the NAC process, procedures at RTPs are less standardized, which caused concerns for candidates. Although beyond the responsibilities of the NAC, there is great variation in what RTPs are doing regarding selection following receipt of candidates’ rankings. That each RTP conducts secondary processes including re-interviewing in various ways and base decisions on a variety of contributing factors might detract from the NAC’s social validity, if the candidates’ perception of fairness decreases as the selection process progresses. Individual RTPs are encouraged to embrace the notion of social validity [25] in their individual procedures.

### Feedback

Feedback has been defined as “specific information about the comparison between a trainee’s observed performance and a standard, given with the intent to improve the trainee’s performance” [39]. Candidates reported that although they would have liked to, they did not receive any formative feedback on their performance. They also provided no indication of expecting to receive summative feedback, even if they were successful. There was a considerable wealth of material available from the interview process itself including marks and in depth qualitative comments on their interview performance. However, one consideration is that providing summative feedback may provide an unfair advantage to subsequent applications for the same candidate. Perceptions of fairness have been linked to litigation [17,40], and the question needs to be asked whether or not providing feedback would affect litigation, and what type of feedback should be given, if any.

Regarding formative feedback, Anderson (2011) recommends that candidates be provided with regular opportunities for verbal feedback during testing. Regarding summative feedback, Anderson (2011) suggests that allowing candidates to review their test scores, and the introduction of a standardised appeal process within the selection system, may reduce the possibility of litigation by disgruntled candidates [8]. It should be noted that candidates to have the opportunity to ask for feedback on their performance, though they cannot review their assessment papers. In the Parliament of the Commonwealth of Australia, 2012 report on the inquiry into registration processes and support for International Medical Graduates, it was recommended that the Australian Medical Council provide a “detailed level of constructive written feedback for candidates who have undertaken the Australian Medical Council’s Structured Clinical Examination” [41]. It may therefore be timely for all relevant stakeholders to give careful consideration to the issue of feedback in relation to selection into specialty training.

#### Implications

Social validity theory offers a useful theoretical framework for exploring candidates’ perceptions of procedural fairness [28]. The key elements of this theory are related to organisational attractiveness, intentions to recommend the selection process to others, and job acceptance [6,20,42]. However within the healthcare professional literature there are typologies of the validity of assessments which already exist [43], in additional to the Van Der Vleuten notion of utility (1996) [26]. Typically they describe of content, construct, and criterion validities, with criterion split into concurrent and predictive depending on the timing of the studies. Validity can be describe as a unitary concept, which describes the reasonableness of any assessment strategy and how this is demonstrated [43]. Accordingly, medical educators would need to determine the reasonableness of a selection strategy that has located selection within a gatekeeping role in preparing a workforce to provide safe, just and effective health care provision. In developing applications of organisational justice within selection into postgraduate training, Patterson et al. [44] have introduced the notion of political validity. This describes the extent to which varied groups of interested stakeholders feel that any selection system meets their basic requirements and will yield valid results before the assessment is even administered and often without ever seeing the assessment itself or any evidence of its psychometric soundness [45]. The question arises as to what extent notions of social validity [25], with its focus on the candidates’ perspective, fit in with existing frameworks of validity such as Downing’s [43] as they relate to selection.

In our study, the least met aspect of social validity of the NAC approach, was candidates’ minimal expectation of getting feedback. From an institution perspective, the issue of whether to give feedback within the selection processes for a limited number of places is problematic [46]. Complaints are more likely where unsuccessful candidates are handled poorly. However, institutions should have little to fear if they have met the criteria for social validity in their recruitment process. There is also the issue of whether assessors are competent to give good written narrative feedback after a complex performance assessment like the MMI [47]. Selection committees could learn much from the work done in the work based assessment sphere [48], where there has been a call to improve training around the giving of feedback which is specific to the assessee’s professional development. However, the purpose of feedback in assessment centres has to be clearly defined. For successful candidates feedback would be helpful for training, whereas for unsuccessful candidates it may be helpful for re-applying or considering other career choices.

#### Limitations of the study

We believe this is the first study of its kind exploring the experiences of candidates and participants about a National Assessment Centre approach in which the non-cognitive tests of an MMI and an SJT have been used to determine suitability for specialist training. Inevitably in qualitative studies, the context of the field of study may not be generalisable to other settings. The immediacy of data collection to the NAC process added to the authenticity of data collected but limited what and how much data it was possible to collect from candidates without adding a burden to what was already a stressful high stakes selection process. We accept that in quantifying thematic prevalence we had less than 50% of responders, but still claim sufficient representedness of the whole cohort.

Due to the questionnaire being anonymous, we were unable to link feedback of the process with candidate performance.

## Conclusion

During any job selection process, not only is the organisation assessing the candidates, but the candidates are also assessing the organisation. We used the Social Validity Theory as a framework with which to interpret candidates’ perceptions of fairness of the selection process. In the context of a national selection process candidates generally felt; treated with dignity and respect, that they were involved and participatory, selection processes were fairly transparent and unambiguous, but were equivocal about the likelihood of receiving feedback on their performance. However, focus on the candidate experience throughout an organisation’s selection process may provide benefits to both candidates and the organisation, regardless of whether or not candidates secured the job. Social Validity Theory is a useful addition to the methods for demonstrating the reasonableness of any selection system.
